# Prognostic Impact of Ki-67 Change in Locally Advanced and Early Breast Cancer after Neoadjuvant Chemotherapy: A Single Institution Experience

**DOI:** 10.1155/2021/5548252

**Published:** 2021-05-04

**Authors:** Mirco Pistelli, Filippo Merloni, Sonia Crocetti, Laura Scortichini, Laura Tassone, Luca Cantini, Veronica Agostinelli, Lucia Bastianelli, Agnese Savini, Rossana Berardi

**Affiliations:** Clinica Oncologica e Centro Regionale di Genetica Oncologica, Università Politecnica delle Marche, AOU Ospedali Riuniti, Ancona (AN) 60126, Marche, Italy

## Abstract

Systemic neoadjuvant chemotherapy (NCT) is a standard treatment for locally advanced breast cancer (LABC) and for selected early breast cancer (EBC). In these settings, the prognostic and predictive role of Ki-67 before and after NCT is unclear. The aim of our study was to investigate the prognostic role of Ki-67 change in patients not achieving pathological complete response (pCR). We retrospectively analyzed data of patients who did not achieve pCR assessing Ki-67 expression pre- and post-NCT. We stratified three groups: high reduction (>20%), low reduction (1–20%), and no reduction in Ki-67. These groups were correlated with clinical and pathological data by *χ*2 test. We estimated disease-free survival (DFS) and overall survival (OS) using Kaplan–Meier method, and we adopted univariate and multivariate Cox proportional hazard models. We selected 82 patients from a database of 143 patients, excluding those who were metastatic at diagnosis, achieved pCR, or lack data regarding Ki-67. Median age at diagnosis was 54 years (range 30–75); 51 patients were Luminal B, 10 human epidermal growth factor receptor 2 (HER-2) enriched, and 21 triple negative. A significant correlation between high Ki-67 reduction and luminal B HER-2-negative subtype was observed (*p* = 0,0035). The change in Ki-67 was significantly associated with DFS (*p* = 0,0596) and OS (*p* = 0,0120), also at multivariate analysis (*p* = 0,0256 for DFS; *p* = 0,0093 for OS). In particular, as compared to patients with low/no reduction of Ki-67, those with high Ki-67 reduction (>20%) after NCT showed better survival (60% vs. 56% vs. 83% after 5 years from diagnosis, respectively; *p* = 0.01). In conclusion, in our study, Ki-67 change showed a significant prognostic role in breast cancer patients treated with NCT who did not achieve pCR. Crucially, Ki-67 < 20% identifies a high-risk population that may be eligible for clinical trials with novel therapeutic interventions in adjuvant setting.

## 1. Introduction

Systemic neoadjuvant therapy (NCT) is the current standard treatment for locally advanced breast cancer (LABC) and for selected early breast cancer (EBC). Indeed, the main purposes of NCT are both the conversion of unresectable cancer into being operable and the downstaging of large ones in favor of breast-conserving surgery rather than mastectomy. Among different breast cancer subtypes, human epidermal growth factor receptor 2 (HER-2) positive, luminal B/HER-2-negative, and, especially, triple-negative tumors are more responsive to NCT with a higher percentage of pathological complete response (pCR) as discussed elsewhere [1]. The achievement of pCR has been widely associated with better prognosis, and it is demonstrated that triple-negative patients with pCR have comparable survival to non-triple-negative patients as discussed by Von Minckwitz et al. [2]. Therefore, the higher risk of recurrence in patients with residual invasive disease at surgery prompted clinical studies investigating a potential escalation of postsurgical systemic therapy. The CREATE-X trial (2017) demonstrated a benefit in terms of disease-free survival (DFS) and overall survival (OS) with the addition of capecitabine to standard adjuvant therapy in HER-2-negative patients with residual invasive breast cancer after NCT as discussed elsewhere [3]. The recent KATHERINE trial involving HER-2 positive EBC population without pCR after NCT showed a 50% lower risk of recurrence in patients treated with adjuvant TDM-1 than those treated with standard trastuzumab as discussed by Von Minckwitz et al. [4]. However, patients who do not achieve pCR are heterogeneous group, and potential side effects and increased costs linked to a more aggressive postsurgery treatment prompt the detection of prognostic and predictive biomarkers that can identify patients more likely to benefit from escalated adjuvant strategies. Ki-67 is a nuclear non-histone protein associated with cellular proliferation, present in all active phases of cell cycle, except the G0 phase as discussed elsewhere [5]. The Ki-67 index is defined as the percentage of positively stained cells among the total number of malignant cells scored in tumor tissue. The Ki-67 index is widely accepted in EBC as an independent prognostic factor. High levels of Ki-67 are significantly associated with worse OS and DFS as discussed elsewhere [6, 7], and it represents a crucial factor for adjuvant treatment selection in clinical practice, for example, in the choice of addition of chemotherapy to hormone therapy in estrogen-receptor (ER) and progesterone-receptor (PgR) expressing breast cancer. However, in the neoadjuvant setting, the prognostic role of Ki-67 remains controversial and is not currently determinant in postsurgery therapeutic decisions. The neoadjuvant setting offers the opportunity to evaluate three different Ki-67 parameters: pretreatment Ki-67, post-treatment Ki-67, and Ki-67 change between them. The role of pretreatment Ki-67 index in predicting the achievement of pCR is well established as discussed elsewhere [8, 9], but results concerning its prognostic validity are controversial as discussed elsewhere [10–13]. High post-treatment Ki-67 has been strongly correlated with worse OS and DFS as discussed elsewhere [14, 15], even though some studies did not substantiate this hypothesis. The change of Ki-67 index due to NCT has been related to both OS and DFS as discussed by Li et al. [16]. This parameter has the advantage of including pre- and post-treatment Ki-67 values simultaneously, and, furthermore, it could bring information about tumor sensitivity to NCT. However, data regarding the prognostic role of Ki-67 change are limited, and there is not a standardized cut-off value. The aim of our study was to investigate the prognostic role of Ki-67 change after NCT in a subset of LABC and EBC, which did not achieve pCR.

## 2. Methods

### 2.1. Patients

We retrospectively identified patients with histological diagnosis of LABC and EBC who underwent taxane and anthracycline-based NCT at our Institution (Università Politecnica delle Marche – Ospedali Riuniti di Ancona) between 2005 and 2017, followed by surgery with radical intent. Trastuzumab was added to NCT based on HER-2 status. Patients had adjuvant treatment with trastuzumab (until the completion of one-year treatment) and/or hormonal therapy depending on HER-2 and hormonal receptors (HR) expression. Follow-up included physical examination, Carcinoembryonic Antigen (CEA) and Cancer Antigen 15.3 (CA 15.3) dosage, annual mammography, and mammary ultrasound. Eligibility criteria included a histological diagnosis of breast cancer, age ≥18 years, Eastern Cooperative Oncology Group performance status 0–2, failure to achieve pCR (defined as the absence of invasive residual disease in breast and lymph nodes), and assessment of Ki-67 expression by immunohistochemistry (IHC) in pre- and post-NCT tissue samples. The study obtained the approval of the Department of Medical Oncology, AO Ospedali Riuniti, Ancona. According to the Italian legislation, since data were retrospectively collected, and there was not direct patient involvement, the ethical approval and patients consent for the study were not required (Official Gazette No. 72 of March 26, 2012). We reviewed medical record to find data on patient's characteristics and clinical-pathological features: age, body mass index (BMI), clinical stage defined on the basis of the version of the American Joint Committee on Cancer (AJCC) Staging Manual validated at the time of the diagnosis, histotype (ductal, lobular, and mixed), grade (determined on pretreatment tissue sample), lymphovascular invasion, ER and PgR status, HER-2 status, and Ki-67 expression in pre- and post-NCT tissue samples (shown in Table 1). ER and PgR≥1% were considered positive, and HER-2 status was estimated by IHC using a semiquantitative score (from 0 to 3+). HER-2 positivity was defined by a 3+ score, while score 1+ was considered negative. In case of 2+ score by IHC, HER-2 status was determined by fluorescence in situ hybridization (FISH). According to the expression of ER, PgR, and HER-2, we identified four phenotypes: luminal B/HER-2-negative (HER-2-negative, ER-positive, any PgR, any Ki67), luminal B/HER-2 positive (HER-2 positive, ER-positive, any PgR, any Ki-67), HER-2 enriched (HER-2 positive, ER and PgR negative), and triple negative defined as lack of expression of HER-2, ER, and PgR. Rare histological types of triple-negative breast cancer (TNBC) were excluded from this study. For the aim of our analysis, patients were divided into three groups according to Ki-67 change after NCT: patients with high reduction (>20%), low reduction (1–20%), and no reduction in Ki-67 index.

### 2.2. Statistical Analysis

We selected OS as primary endpoint and DFS as secondary endpoint. OS was defined as the interval between histological diagnosis to death or last follow-up. DFS was defined as the interval between diagnosis of breast cancer to the first failure, including locoregional and/or distant relapse, second primary or death. Patients who were not reported as dead at the time of the analysis were censored at the date they were last known to be alive. Survival distribution was estimated by the Kaplan–Meyer method and was compared between the strata through log-rank test. The association between categorical variables was estimated by Chi square test. The Cox multivariate proportional hazard regression model was used to evaluate the effects of the prognostic factors on survival. Hazard ratios and 95% confidence intervals (CIs) were estimated from regression coefficients. The estimated risk of death or recurrence was adjusted for age at the time of diagnosis, AJCC stage, lymphovascular invasion, and breast cancer phenotype. Significance level in the univariate model for inclusion in the multivariate final model was set at a 0.05 as discussed elsewhere [17, 18]. All other significance levels were set at a 0.05 value, and all P values were two-sided. Statistical analysis was performed with the MedCalc package (MedCalc® v9.4.2.0 Software, Ostend, Belgium).

## 3. Results

**Figure 1:**
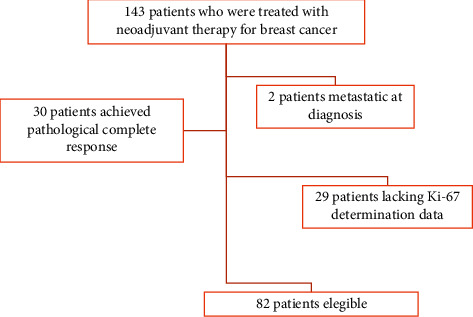
We identified 143 patients treated with neoadjuvant therapy for breast cancer; 82 patients were eligible for the analysis.

82 patients treated with NCT (with or without targeted therapy) were selected from a database of 143 patients, excluding those who were metastatic at the diagnosis, those who achieved pCR, and those lacking data regarding Ki-67 determination (shown in Figure 1).

Median age was 54 years (range 30–75 years), and 46 patients (56,1%) were in postmenopausal status. BMI, assessed before treatment administration, was above 30 kg/mq in 12 patients (14,6%), between 25 and 29,9 kg/mq and under 24,9 kg/mq in 35,5% and 48,7%, respectively. Clinical stage (AJCC) was I in 28,1% of patients, II in 47,5%, and III in 20,8%. Lymphovascular invasion was present in 43,6% of patients. The most observed tumor grade, determined in pre-NCT tissue sample, was G3 (72%). 35 patients (42,7%) were Luminal B/HER-2−, 19,5%, Luminal B/HER-2 +, 12,2%, HER-2 enriched, and 25,6%, triple negative. Patients with a high Ki-67 reduction (>20%) were 43,8%; 28,1%, patients had a Ki-67 reduction between 1% and 20%, and 28,1%, had no reduction (shown in Table 1).

**Figure 2:**
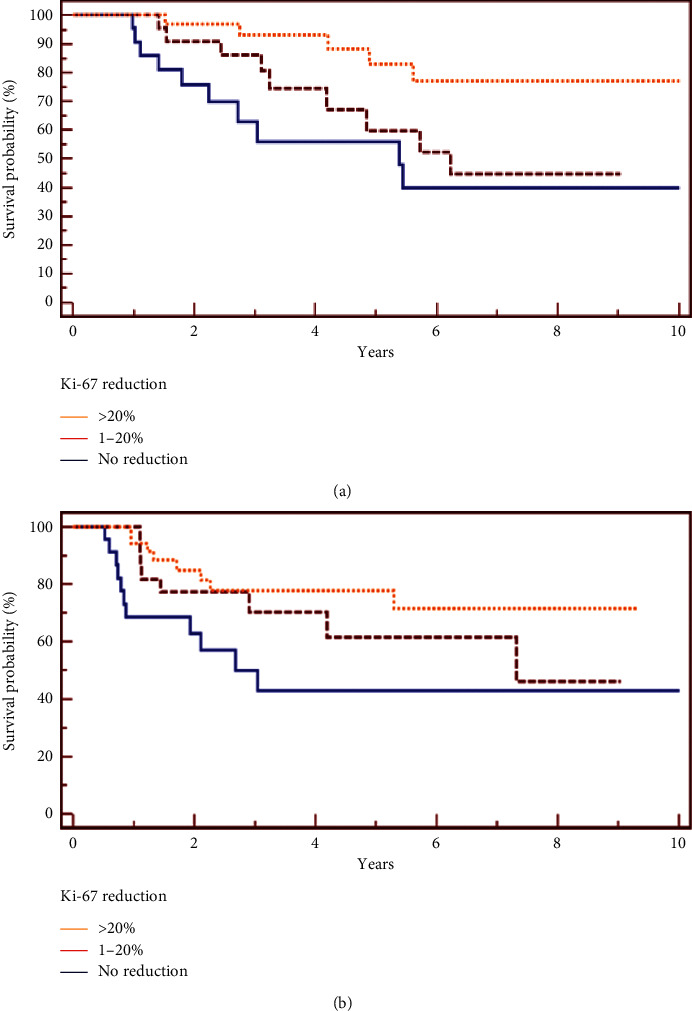
(a) Ki-67 reduction associated with Overall Survival (*p*=0,0120); (b) Ki-67 reduction associated with Disease-Free Survival (*p*=0,0596).

A significant correlation among Ki-67 change and molecular subtype was observed (*p* = 0,0035). In particular, patients with a high reduction in Ki-67 were more likely Luminal B/HER-2− (61,2%). There was no significant correlation between Ki-67 change subgroups and other clinical-pathological factors, including age (*p* = 0,83), BMI (*p* = 0,33), histotype (*p* = 0,65), grading (*p* = 0,12), lymphovascular invasion (*p* = 0,25), and clinical stage (*p* = 0,71) (shown in Table 1). The median follow-up time was 68, 7 months (range 7, 2–141 months). Death and recurrence were documented in 24 and 28 patients, respectively. Univariate analysis revealed a significant association between Ki-67 change subgroups and OS (*p* = 0,0120). In particular, the 5-year OS was 83% for patients with high Ki-67 reduction, compared to 60% and 56% for low reduction and no reduction subgroups, respectively. The median OS for high Ki-67 reduction patients was not reached. Median OS for low reduction and no reduction/increase subgroups was 74,4 and 63,6 months, respectively (shown in Figure 2(a)).

The Cox proportional multivariate hazard model showed that Ki-67 change was independently associated with OS (95% CI 0,2962–0,8406, hazard ratio = 0, 4990, *p*=0,0093) after adjusting for covariates (age, tumor molecular subtype, clinical stage, and lymphovascular invasion) (shown in Table 2).

No statistically significant correlation between Ki-67 change subgroups and OS at univariate analysis was observed when we considered Luminal B/HER-2− (*p*=0,15), HER-2 + (*p*=0,3), and triple-negative (*p*=0,1) patients separately. High and low Ki-67 reduction subgroups were then compared, not considering no reduction subgroup. Univariate analysis showed that OS was significantly different between high and low reduction subgroups (95% CI 1,0529–9,1877, hazard ratio=2,9687, *p*=0,04) with a favorable prognosis for patients with a decrease in Ki-67 index > 20%. In univariate analysis, we found an association of borderline significance among Ki-67 change subgroups and DFS (*p*=0,0596), with fewer recurrence for patients with high Ki-67 reduction (shown in Figure 2(b)), which was confirmed in multivariate analysis (95% CI 0,3589–0,93, hazard ratio = 0,5788, *p*=0,0256) after adjusting for covariates (age, tumor molecular subtype, clinical stage, and lymphovascular invasion) (shown in Table 3).

When Ki-67 change subgroups were correlated at univariate analysis with DFS in different molecular subtypes, no statistically significant results were observed (*p*=0,94 for Luminal B/HER-2−; *p*=0,49 for HER-2+; *p*=0,08 for triple negative).

## 4. Discussion

It is well established that pCR after NCT is a surrogate of favorable long-term outcomes as discussed elsewhere [1, 19, 20]. However, BC patients who do not achieve pCR represent a heterogeneous group with different prognoses. A recent phase 3 trial demonstrated a benefit by adding Capecitabine to standard adjuvant therapy in a population of HER-2 patients with residual invasive breast cancer as discussed by Masuda et al. [3]. Similarly, adjuvant TDM-1 improved outcomes compared to trastuzumab for HER-2 + BC patients without pCR after NCT as discussed by Von Minckwitz et al. [4]. Furthermore, there are many studies in progress investigating the role of adjuvant anti-PD1/PD-L1 therapy in this setting (NCT02926196). So, validated biomarkers are needed to better predict prognosis and to tailor post-surgery treatment decisions as discussed elsewhere [21, 22]. Since changes in proliferation anticipate changes in tumor growth rate, Ki67 change might serve as a valuable prognostic marker for patients who do not achieve pCR. However, evidence is still controversial, and Ki-67 is not currently used in post-surgery therapeutic decisions. Most data are derived from retrospective studies, and there is no agreement regarding the selected cut-off points, which were arbitrarily established as discussed by Cabrera‐Galeana et al. [23]. Most of the previous literature focused on the prognostic role of pretreatment or post-treatment Ki-67 in the neoadjuvant setting of BC as discussed elsewhere [16]. Looking at the change of Ki-67 due to neoadjuvant treatment seems to be more dynamic and informative as discussed elsewhere [24], as it could also provide information about tumor sensitivity to chemotherapy and targeted therapy. The greater the reduction of Ki-67, the greater the chance that the NCT regimen might be effective for these patients and could be used as adjuvant or palliative treatment. Conversely, low reduction or no change in Ki-67 may help identify NCT regimens that are less effective and prompt different adjuvant chemotherapy regimens to improve survival in those patients. After dividing patients into three groups according to the Ki-67 change after NCT, we demonstrated that Ki-67 change was independently associated with OS and DFS. In particular, patients with high Ki-67 reduction (>20%) had a lower risk of death (95% CI 0,2962–0,8406, hazard ratio=0,4990, *p* = 0,0093) and relapse (95% CI 0,3589–0,93, hazard ratio=0,5788, *p* = 0,0256), even taking into account other variables known to influence prognosis. A previous meta-analysis including six studies on this topic showed that low reduction, no reduction, or increase was associated with worse DFS (HR: 2.13; 95% CI: 1.51–3.02; I2 = 42%) as discussed elsewhere [16]. Among these studies, only two reported the relationship between change of Ki-67 and OS. In the first one, decreased Ki‐67 rate after NCT was associated with survival (*p* = 0.024) as discussed elsewhere [24, 25]. In the second one, Montagna et al. showed that the decrease of Ki-67 to <20% at final surgery was associated with better DFS (HR 0.52; 95% CI 0.40–0.68 *p* < 0.0001) and OS (HR 0.45; 95% CI 0.32–0.64, *p* < 0.0001) [24, 25]. A recent paper from Cabrera-Galeana and colleagues reported that no reduction of Ki67 significantly increased the risk of recurrence and death by 3.39 (95% confidence interval [CI] 1.8–6.37) and 7.03 (95% CI 2.6–18.7) at multivariate analysis, especially for luminal B subgroup, as discussed by Cabrera-Galeana et al. [23]. In our cohort, no significant correlation between Ki-67 change in each molecular subtypes and OS and DFS at univariate analysis was observed, when we considered Luminal B/HER-2−, HER-2 + and triple-negative patients separately probably due to the small sample size of our study. In our analysis, we also analyzed the absolute change of Ki-67 after NCT. Interestingly, OS was significantly different between high and low reduction subgroups (95% CI 1, 0529–9,1877, hazard ratio = 2,9687, *p* = 0,04) with a favorable prognosis for patients with a decrease in Ki-67 index ≥ 20%. This is contrary to what was reported by Matsubara et al. [26] who showed that patients experiencing 80% reduction in Ki-67 or more had a similar DFS to those experiencing less. They concluded that only the increase of Ki-67 was an independent prognostic factor for DFS. The observed difference might be explained by the fact that Matsubara et al. analyzed the percentage change, rather than the absolute. A reduction from 80% pre-NCT to 40% is probably way more significant than a reduction from 20% to 10%, as it reflects a greater tumor sensitivity to NCT, and the percentage change does not consider this difference. In addition, Matsubara et al. reported 5-year DFS as the primary outcome, which is known to represent a less robust endpoint than OS. Our analysis is hampered by the retrospective nature and the small sample size, and no direct conclusions can be inferred. In addition, there are still concerns about Ki-67 reproducibility, because of interobserver variations that may produce different results. However, patient characteristics and NCT administered were extremely homogenous across subgroups, thus strengthening our results.

## 5. Conclusions

Our study suggests potential cut-off of Ki-67 change, which could be easily used in clinical practice. If prospectively validated in larger cohorts, Ki67 change might serve as a valuable biomarker for BC patients who do not achieve a pCR. Finally, we propose that patients with low Ki-67 change after NCT be treated with escalated adjuvant strategies or be elected for clinical trials with novel therapeutic interventions in adjuvant setting.

## Figures and Tables

**Table 1:** Patients' characteristics and Chi square test results.

Characteristics	No. of patients (%)	No. of no Ki-67 reduction or increase (%)	No. of Ki-67 1–20% reduction (%)	No. of Ki-67 >20% reduction (%)	*p*
—	82 (100)	23 (28.1)	23 (28.1)	36 (43.8)	—
Age (years)					
<50	36 (43.9)	9 (39.1)	11(47.8)	16 (44.4)	0.8350
>50	46(56.1)	14 (60.9)	12 (52.2)	20 (55.6)	

BMI					
<25	40 (48.8)	8 (34.7)	15 (65.2)	17 (47.2)	0.3285
25–30	30 (36.6)	10 (43.5)	6 (26.1)	14 (38.9)	
>30	12 (14.6)	5 (21.8)	2 (8.7)	5 (13.9)	

Histotype					
Ductal	67 (81.7)	18 (78.3)	18 (78.3)	31 (86.1)	0.6526
Lobular	9 (11.0)	3 (13.0)	4 (17.4)	2 (5.6)	
Mixed	6 (7.3)	2 (8.7)	1 (4.3)	3 (8.3)	

Grade					
2	23 (28.0)	4 (17.4)	10 (43.5)	9 (25.0)	0.1241
3	59 (72.0)	19 (82.6)	13 (56.5)	27 (75.0)	

Lymphovascular invasion					
Absent	46 (56.4)	10 (43.5)	16 (69.6)	20 (55.6)	0.2542
Present	36 (43.6)	13(56.5)	7 (30.4)	16 (44.4)	

Breast cancer phenotype					
Luminal B HER-2 (−)	35 (42.7)	5 (21.8)	8 (34.8)	22 (61.2)	0.0035
Luminal B HER-2 (+)	16 (19.5)	3 (13.0)	9 (39.1)	4 (11.1)	
HER-2 (+)	10 (12.2)	6 (26.1)	1 (4.3)	3 (8.3)	
Triple negative	21 (25.6)	9 (39.1)	5 (21.8)	7 (19.4)	

Clinical stage					
1	23 (28.1)	7 (30.5)	6 (26.1)	10 (27.8)	0.7102
2	39 (47.5)	11 (47.8)	12 (52.1)	16 (44.4)	
3	17 (20.8)	3 (13.0)	5 (21.8)	9 (25.0)	
4	3 (3.6)	2 (8.7)	0 (0.0)	1 (2.8)	

BMI, body mass index; HER-2, Human Epidermal Growth Factor Receptor 2.

**Table 2:** Multivariate analysis (OS).

Variable	*p*	HR	95% CI
Age (≤50 vs. >50)	0,9238	0,9586	0,4052 to 2,2678
Breast cancer molecular subtype	0,0028	1,7612	1,2174 to 2,5479
Clinical stage (II vs. III)	0,1418	1,5616	0,8643 to 2,8216
Lymphovascular invasion (absent vs. present)	0,2583	1,7129	0,6769 to 4,3347
Ki-67 change (pre- vs. post-NCT)	0,0093	0,4990	0,2962 to 0,8406

OS, overall survival; HR, hazard ratio; CI, confidence interval; NCT, neoadjuvant chemotherapy.

**Table 3:** Multivariate analysis (DFS).

Variable	*p*	HR	95% CI
Age (≤50 vs. >50)	0,6985	1,1786	0,5152 to 2,6961
Breast cancer molecular subtype	0,0348	1,4441	1,0283 to 2,0281
Clinical stage (II vs. III)	0,6204	1,1359	0,6878 to 1,8758
Lymphovascular invasion (absent vs. present)	0,3984	1,4311	0,6255 to 3,2745
Ki-67 change (pre- vs. post-NCT)	0,0256	0,5788	0,3589 to 0,9333

DFS, disease-free survival; HR, hazard ratio; CI, confidence interval; NCT, neoadjuvant chemotherapy.

## Data Availability

The datasets used and/or analyzed during the present study are available from the corresponding author.
